# Etiology of Diarrhea Among Hospitalized Children in Blantyre, Malawi, Following Rotavirus Vaccine Introduction: A Case-Control Study

**DOI:** 10.1093/infdis/jiz084

**Published:** 2019-02-28

**Authors:** Miren Iturriza-Gómara, Khuzwayo C Jere, Daniel Hungerford, Naor Bar-Zeev, Kayoko Shioda, Oscar Kanjerwa, Eric R Houpt, Darwin J Operario, Richard Wachepa, Louisa Pollock, Aisleen Bennett, Virginia E Pitzer, Nigel A Cunliffe

**Affiliations:** 1 Centre for Global Vaccine Research, Institute of Infection and Global Health, University of Liverpool, United Kingdom; 2 Malawi-Liverpool-Wellcome Trust Clinical Research Programme, Blantyre; 3 Department of Medical Laboratory Sciences, College of Medicine, University of Malawi, Blantyre; 4 International Vaccine Access Center, Johns Hopkins Bloomberg School of Public Health, Baltimore, Maryland; 5 Department of Epidemiology of Microbial Diseases, Yale School of Public Health, Yale University, New Haven, Connecticut; 6 Division of Infectious Diseases and International Health, University of Virginia, Charlottesville

**Keywords:** Gastroenteritis, diarrhea, children, PCR, rotavirus, case-control, Malawi

## Abstract

Despite rotavirus vaccination, diarrhea remains a leading cause of child mortality. We collected stool specimens from 684 children <5 years of age hospitalized with diarrhea (cases) and 527 asymptomatic community controls for 4 years after rotavirus vaccine introduction in Malawi. Specimens were tested for 29 pathogens, using polymerase chain reaction analysis. Three or more pathogens were detected in 71% of cases and 48% of controls. Pathogens significantly associated with diarrhea included rotavirus (in 34.7% of cases and 1.5% of controls), enteric adenovirus (in 29.1% and 2.7%, respectively), *Cryptosporidium* (in 27.8% and 8.2%, respectively), heat-stable enterotoxin-producing *Escherichia coli* (in 21.2% and 8.5%, respectively), typical enteropathogenic *E. coli* (in 18.0% and 8.3%, respectively), and *Shigella*/enteroinvasive *E. coli* (in 15.8% and 5.7%, respectively). Additional interventions are required to prevent diarrhea due to rotavirus and other common causal pathogens.

## Teaser

Despite health gains achieved through rotavirus vaccination, rotavirus remains the most common cause of severe, hospitalized diarrhea among children in Malawi. There is a pressing need to identify strategies to improve rotavirus vaccine performance in low-income countries with a high disease burden.

The introduction of rotavirus vaccines into childhood immunization programs across Africa has reduced the incidence of rotavirus-associated hospitalizations, with a recent population-based study from Malawi providing the first evidence of the vaccines’ impact on infant diarrheal deaths [1]. However, the contribution of bacterial, parasitic, and other viral causes of diarrhea among rotavirus-vaccinated African populations is not well understood [2, 3].

Malawi is a low-income country in sub-Saharan Africa. Among adults, it has one of the highest prevalence rates of human immunodeficiency virus (HIV) infection, with or without AIDS, globally; 17.8% of women 15–49 years of age living in urban Malawi are infected with HIV [4]. In children <5 years of age, the mortality rate is 55 cases/1000 live births, with a high prevalence of stunting (37%).

Monovalent human rotavirus vaccine (Rotarix) was introduced into Malawi’s national immunization schedule in October 2012. At the Malawi-Liverpool-Wellcome Trust Clinical Research Programme in Blantyre, Malawi, we have examined the effectiveness of Rotarix against rotavirus diarrhea requiring hospitalization [5]. Our diarrhea surveillance platform is centered on a large urban hospital (Queen Elizabeth Central Hospital [QECH]) and includes collection of clinical, demographic, and socioeconomic data, combined with documentation of rotavirus vaccine status and stool specimen collection.

We aimed to understand the etiology of severe acute childhood gastroenteritis following rotavirus vaccine introduction in Malawi. We therefore undertook a case-control study to examine the prevalence of enteric pathogens among hospitalized children <5 years of age with diarrhea, compared with findings for diarrhea-free children in the community.

## METHODS

### Study Setting

The study was conducted in Blantyre, a district in southern Malawi with a population of approximately 1.3 million people. QECH provides free healthcare to residents of urban and rural areas of Blantyre District and is the only free, government-run inpatient referral center in southern Malawi. Monovalent rotavirus vaccine is administered according to the World Health Organization–recommended schedule of 6 and 10 weeks of age. Vaccine coverage increased rapidly following introduction and has exceeded 90% among age-eligible children since 2014 [1, 6].

### Symptomatic Hospitalized Cases

Subject enrollment and specimen collection were undertaken as part of our existing research program [5]. Stool specimens were collected from November 2012 to December 2015 from children <5 years of age with acute gastroenteritis who were admitted to QECH (hereafter, “cases”). Acute gastroenteritis was defined as the passage of ≥3 loose stools within a 24-hour period commencing <14 days before enrollment. We obtained demographic, clinical, and anthropometric data through parental interview, physical examination, and review of medical notes. Maternal HIV testing was undertaken according to national guidelines, as previously reported [5]. Disease severity was measured using the Vesikari scoring system; a score of ≥11 (out of 20) indicates severe disease.

### Asymptomatic Community Controls

Stool specimens were collected from children in the community who reported no diarrhea for at least 7 days (hereafter, “controls”). These composed a random sample of community controls recruited between April 2013 and December 2016 into case-control studies of rotavirus vaccine effectiveness, in which controls were matched by age and district of residence to cases with confirmed rotavirus diarrhea [5, 6].

### Laboratory Testing

Fecal specimens were stored at −80^º^C. We tested for 29 different enteric pathogens in fecal specimens, using a 384-well singleplex real-time polymerase chain reaction (PCR) analysis platform, the enteric Taqman Array Cards (TAC) [7]. The TAC method performance has been previously described; samples were classified as pathogen positive at a cycle threshold of <35 [7]. Detection above this threshold is not reproducible and is unlikely to be clinically significant [7].

### Analysis

Since the introduction of rotavirus vaccination, norovirus has gained increasing recognition as a significant pathogen associated with diarrhea in children [8]. We therefore based our sample size calculation on the norovirus prevalence. Assuming prevalence proportions of 10% among hospitalized cases with gastroenteritis and 5% among asymptomatic community controls [9], we calculated that, for 90% power, 621 samples per group were needed to show a significant difference in prevalence between cases and controls (α = 0.05). Pathogen prevalence among cases and controls was compared using the χ^2^ test or the Fisher exact test. Differences between continuous variables were tested using the Student *t* test or the Wilcoxon rank sum test.

Adjusted attributable fractions (AFs) were calculated for pathogens that had a greater prevalence among cases as compared to controls. Using a similar approach to that in the Global Enteric Multicentre Study (GEMS) [10], multivariable logistic regression was used to calculate odds ratios (ORs) for each pathogen detected, adjusting for presence of other pathogens, age in months and month of recruitment. Case or control status was the outcome variable and the predictors were indicator variables representing the presence or absence of each pathogen. We then calculated AFs for pathogens that were significant (*P* < .05) in multivariable logistic regression. Calculation of AFs was as follows:

$$\begin{array}{l} AF_{i}={Prevalence}_{i}\times \left(1-\frac{1}{OR_{i}}\right), \end{array}$$

*AF_i_*, attributable fraction for pathogen *i* among cases

*OR_i_*, adjusted odds ratio for pathogen *i* among cases

where *Prevalence*_*i*_ denotes the prevalence of pathogen *i* among cases. Analyses were performed using R version 3.3.5 (R Development Core Team, Vienna, Austria), with AFs and associated 95% confidence intervals (CIs) calculated using bootstrap resampling in the “attribrisk” package.

### Ethics

Approval was obtained from the Malawi National Health Sciences Research Committee (protocol 837) and from the University of Liverpool Research Ethics Committee (RETH protocol 490).

## RESULTS

### Study Population

A total of 1211 fecal samples were analyzed, comprising 684 samples from cases and 527 samples from controls (Supplementary Table 1). The median age was 10.7 months (interquartile range [IQR], 7.9–15.4 months) and 12 months (IQR, 9.2–18.7 months) for cases and controls, respectively. Rotavirus vaccine status was established for 419 of 451 cases who met the age-based eligibility criterion for vaccination (of whom 97% had received ≥1 dose) and for 375 of 400 controls who met the age-based eligibility criterion (of whom 95% were vaccinated). Maternal HIV status was determined for 665 of 684 cases (97%), of whom 18% were HIV exposed. Mid-upper arm circumference was lower in cases (median, 13.0 cm), compared with controls (15.3 cm). Among cases, 60 (9%) were severely malnourished (mid-upper arm circumference, < 11.5 cm), compared with 2 controls (0.4%). The Vesikari score among cases ranged from mild (score, 1) to very severe (score, 20), with a median score of 13 (IQR, 10–15).

### Pathogen Prevalence

A positive PCR result for at least 1 pathogen was returned for 641 samples (94%) from cases and 390 (74%) from controls (*P* < .001; Supplementary Table 1). Pathogens that were more frequently identified in cases than in controls included, in decreasing order of prevalence (cases/controls), rotavirus (34.7% of cases vs 1.5% of controls), adenovirus 40/41 (29.1% vs 2.7%), *Cryptosporidium* (27.8% vs 8.2%), heat-stable enterotoxin-producing *Escherichia coli* (ST-ETEC; 21.2% vs 8.5%), typical enteropathogenic *E. coli* (EPEC; 18.0% vs 8.3%), *Shigella/*enteroinvasive *E. coli* (EIEC; 15.8% vs 5.7%)*, Salmonella* Typhimurium (2.3% vs 0.4%), and *Vibrio cholerae* (1.3% vs 0%; Table 1). Pathogen prevalence proportions stratified by age group are presented in Supplementary Table 2. *Giardia* was less frequently detected in cases, compared with controls (7.3% vs 13.9%). Enteroaggregative *E. coli* (EAEC), *Campylobacter*, and norovirus were frequently detected in both cases and controls. Pathogens with the highest AFs were rotavirus, adenovirus 40/41, and *Cryptosporidium* (Table 1). Median Vesikari scores stratified by pathogen ranged from 11 to 14.5 (Supplementary Figure 1).

**Table 1.** Prevalence and Attributable Fractions (AFs) for Enteric Pathogens Among Children Hospitalized With Diarrheal Disease (Cases) and Asymptomatic Children From the Community (Controls)

Pathogen	Cases, No. (%) (n = 684)	Controls, No. (%) (n = 527)	*P*	Adjusted AF ± SE, %	95% CI
EAEC	354 (51.8)	252 (47.8)	.193	…	…
Rotavirus	237 (34.6)	8 (1.5)	<.001	34.2 ± 0.018	31.1–38.0
Adenovirus 40/41	199 (29.1)	14 (2.7)	<.001	27.7 ± 0.018	24.5–31.4
*Cryptosporidium*	190 (27.8)	43 (8.2)	<.001	22.3 ± 0.023	18.2–27.2
ST-ETEC	145 (21.2)	45 (8.5)	<.001	12.7 ± 0.030	7.2–18.1
Typical EPEC	123 (18.0)	44 (8.3)	<.001	6.6 ± 0.037	0–12.2
Any *Campylobacter*	113 (16.5)	102 (19.4)	.229	…	…
Shigella/EIEC	108 (15.8)	30 (5.7)	<.001	10.8 ± 0.021	6.5–14.9
Norovirus	83 (12.1)	45 (8.5)	.054	7.0 ± 0.019	2.8–10.6
**A**typical EPEC	77 (11.3)	80 (15.2)	.054	…	…
LT-ETEC	68 (9.9)	71 (13.5)	.069	…	…
Sapovirus	64 (9.4)	34 (6.5)	.083	…	…
*Giardia*	50 (7.3)	73 (13.9)	<.001	…	…
*Enterocytozoon bieneusi*	31 (4.5)	21 (4.0)	.747	…	…
**Any *S*** *almonella*	30 (4.4)	5 (0.9)	.001	…	…
Aeromonas	27 (3.9)	10 (1.9)	.059	…	…
*Salmonella* Typhimurium	16 (2.3)	2 (0.4)	.007	1.8 ± 0.009	.0–3.2
*Encephalitozoon intestinalis*	15 (2.2)	20 (3.8)	.14	…	…
*Cyclospora*	13 (1.9)	4 (0.8)	.138	…	…
Astrovirus	12 (1.8)	13 (2.5)	.509	…	…
*Entamoeba histolytica*	10 (1.5)	8 (1.5)	1	…	…
*Vibrio cholerae*	9 (1.3)	0 (0.0)	.006	…	…
*Salmonella* Typhi	8 (1.2)	2 (0.4)	.201	…	…
*Strongyloides*	7 (1.3)	10 (1.5)	.845	…	…
*Necator*	5 (0.7)	1 (0.2)	.241	…	…
STEC	5 (0.7)	1 (0.2)	.241	…	…
*Ascaris*	4 (0.6)	5 (0.9)	.514	…	…
*Isospora*	3 (0.4)	1 (0.2)	.637	…	…
***Salmonella* E**nteritidis^**a**^	0 (0.0)	0 (0.0)	NA	…	…
*Trichuris* ^**a**^	0 (0.0)	1 (0.2)	.435	…	…
*Ancyclostoma* ^**a**^	0 (0.0)	0 (0.0)	NA	…	…

Pathogens are listed in order of decreasing prevalence among cases.

Abbreviations: CI, confidence interval; EAEC, enteroaggregative *Escherichia coli*; EIEC, enteroinvasive *Escherichia coli*; EPEC, enteropathogenic *Escherichia coli*; LT-ETEC, heat-labile enterotoxin-producing *Escherichia coli*; SE, standard error; STEC, Shiga toxin–producing *Escherichia coli*; ST-ETEC, STh- or STp-producing enterotoxigenic *Escherichia coli*.

^a^ *Ancyclostoma*, *Salmonella* Enteritidis, and *Trichuris* were not included in the analysis because they were not detected in cases or controls.

### Pathogen Distribution, by Age

The median age of *Giardia*-positive cases (15.6 months) was significantly higher than that of *Giardia*-positive controls (11.7 months; *P* = .014). For the following organisms, the median age of pathogen-positive cases was significantly lower than that of pathogen-positive controls: *Campylobacter* (10.9 vs 13.8 months; *P* < .001), LT-ETEC (10.8 vs 12.0 months; *P* = .044), and atypical EPEC (11.3 vs 12.4 months; *P* = .030). There was no difference in age between cases and controls for the remaining pathogens (Supplementary Figure 2).

### Mixed Infections

**Figure 1.**
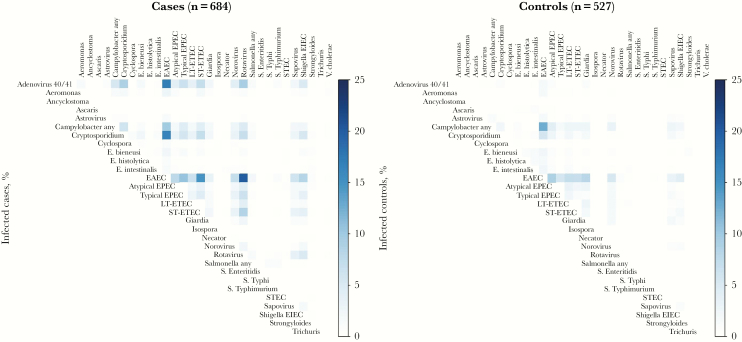
Coinfections among children hospitalized with diarrheal disease (cases) and asymptomatic children from the community (controls). Percentages of cases and controls coinfected with the specified pathogens are shown and were calculated by dividing the number of infections with both pathogen *A* (row) and *B* (column) by the denominator for the relevant group (ie, either total number of cases or total number of controls). EAEC, enteroaggregative *Escherichia coli*; *E. bieneusi*, *Enterocytozoon bieneusi*; *E. histolytica*, *Entamoeba histolytica*; EIEC, enteroinvasive *Escherichia coli*; *E. intestinalis*, *Encephalitozoon intestinalis*; EPEC, enteropathogenic *Escherichia coli*; LT-ETEC, heat-labile enterotoxin-producing *Escherichia coli*; *S.* Enteriditis, *Salmonella* Enteriditis; STEC, Shiga toxin–producing *Escherichia coli*; ST-ETEC, STh- or STp-producing enterotoxigenic *Escherichia coli*; *S.* Typhi, *Salmonella* Typhi; *S.* Typhimurium, *Salmonella* Typhimurium.

Among pathogen-positive samples, a single pathogen was detected in fecal samples from 78 cases (11%) and 42 controls (8%). Cases had a significantly higher number of pathogens (mean [±SD], 3.65 ± 2.11 pathogens) as compared to controls (mean [±SD]. 2.42 ± 1.96 pathogens), with ≥3 pathogens detected in 71% of cases, compared with 48% of controls. Enteroaggregative *E. coli* (EAEC) was identified in 51.8% of cases and 47.8% of controls. The most frequent coinfections involving EAEC were with rotavirus, adenovirus 40/41, *Cryptosporidium,* ST-ETEC, and typical EPEC, among cases, and with *Campylobacter*, atypical EPEC, *Giardia*, and LT-ETEC, among controls (Figure 1).

## Discussion

We have shown that, following rotavirus vaccine introduction in Malawi, rotavirus remains the leading pathogen detected in children <5 years of age hospitalized because of diarrhea. This is in agreement with data from 2 smaller studies, including one, performed in Tanzania, that assessed 146 diarrhea cases in 2015 and another, reported by the Global Rotavirus Surveillance Network, that analyzed a total of 327 samples collected from 10 African countries between 2013 and 2014 [2, 3]. The prevalence of rotavirus shedding in asymptomatic children reported in this study (1.5%) is lower than reported previously in Malawi [11]. This may be the consequence of a high level of rotavirus vaccine coverage, which reduced rotavirus transmission in the community and potentially yielded indirect benefits [6].

Adenovirus 40/41 was significantly associated with hospitalization for diarrheal disease, in contrast with the Tanzanian and Global Rotavirus Surveillance Network studies, which reported a modest AF for this pathogen [2, 3]; however, the AFs calculated by these studies relied on samples from asymptomatic individuals in the community that were collected during the GEMS study, before rotavirus vaccine introduction [10]. Furthermore, pathogen-specific AFs should be interpreted with caution, since the relationship between organism load, disease severity, and duration of shedding are not well defined for pathogens other than rotavirus. Nevertheless, the high prevalence of adenovirus 40/41 among cases, combined with a low prevalence among asymptomatic children, suggests an important role for this pathogen in severe diarrhea in Malawian children. Our data are supported by recent reanalysis of the GEMS and MAL-ED (The Aetiology, Risk Factors, and Interactions of Enteric Infections and Malnutrition and the Consequences for Child Health) studies, which highlighted the importance of adenovirus 40/41 among children with diarrhea [12, 13].

*Cryptosporidium* was frequently associated with hospitalization for diarrhea in Malawian children, with a higher prevalence than reported in studies conducted elsewhere [2, 3, 10, 12, 13]. *Cryptosporidium* diarrhea is associated with 4.2 million disability-adjusted life-years (DALYs) lost globally and a loss of 7.85 million DALYs after accounting for the negative impact it has on growth [14]. These data highlight the need to prioritize effective treatment and prevention of *Cryptosporidium* infection in Malawi and other low-income countries that have high rates of stunting and HIV/AIDS, to reduce the burden of diarrheal disease and its associated long-term health consequences.

We also identified ST-ETEC, typical EPEC, and *Shigella*/EIEC as important pathogens associated with hospitalization for diarrheal disease, in line with previous reports [2, 12, 13]. Other pathogenic *E. coli* were also commonly detected. EAEC was found in nearly half of infants, regardless of the presence of diarrhea, and was frequently identified in coinfections among both cases and controls. Although EAEC does not appear to be associated with acute diarrhea in this or previous studies [2, 3, 10], its presence with ≥2 pathogens may have a negative impact on infant growth [15]. Similarly, despite a lack of association with hospitalization for diarrheal disease, the high prevalence of *Campylobacter* and *Giardia* among asymptomatic children in Malawi may result in growth faltering [16].

In some high-income settings, norovirus has emerged as the leading cause of severe diarrhea in children following rotavirus vaccine introduction [15]. However, we did not document any change in norovirus prevalence since rotavirus vaccine introduction in Malawi; norovirus was identified in 12.1% of children hospitalized with diarrhea, compared with 11.3% in the prevaccine period [16].

Our study has limitations. First, cases were slightly younger than controls; we therefore adjusted for age and month of recruitment in the analyses. We calculated AFs on the basis of the binary presence or absence of pathogens, similar to the original GEMS study [10]. This method differs from those used for the reanalysis of data from the GEMS and MAL-ED studies [13, 16], in which pathogen quantity was included in the model as a quadratic term. We chose not to use this approach, because, unlike longitudinal studies, the duration of pathogen shedding is not quantifiable in case-control studies and because pathogen quantity will be variably associated with disease. A further consideration is our inclusion of cases from November 2012 to March 2013, prior to control recruitment, to achieve the required sample size. In sensitivity analysis, exclusion of the early cases did not significantly alter the point prevalence of any pathogen (data not shown).

Rotavirus remains the most common cause of severe diarrhea requiring hospitalization among children in Malawi, highlighting the importance of identifying strategies to improve rotavirus vaccine performance in this and similar low-income countries with a high disease burden. Adenovirus 40/41, *Cryptosporidium*, ST-ETEC, typical EPEC, and *Shigella*/EIEC are significant additional contributors to the burden of gastrointestinal disease in this population. Public health efforts, including vaccine development, should target these pathogens.

## Supplementary Data

Supplementary materials are available at *The Journal of Infectious Diseases* online. Consisting of data provided by the authors to benefit the reader, the posted materials are not copyedited and are the sole responsibility of the authors, so questions or comments should be addressed to the corresponding author.

jiz084_suppl_Supplementary_Figure_1

jiz084_suppl_Supplementary_Figure_2

jiz084_suppl_Supplementary_Table_1

jiz084_suppl_Supplementary_Table_2

## Notes

***Acknowledgments.*** We thank the collaborating members of the VacSurv Consortium (James Beard, Amelia C. Crampin, Carina King, Sonia Lewycka, Hazzie Mvula, Tambosi Phiri, Jennifer R. Verani, Cynthia G. Whitney, Osamu Nakagomi, Jacqueline E Tate, Umesh Parashar, Rob Heyderman, and Neil French) and the VacSurv and RotaRITE study teams.

***Financial support.*** This work was supported by Takeda Vaccines (investigator-initiated grant 1000452654 to N. A. C., N. B.-Z., and M. I.-G.); the National Institute of Allergy and Infectious Diseases, National Institutes of Health (grant R01AI112970 to V. E. P.); and the Wellcome Trust (programme grant 091909/Z/10/Z to N. A. C., clinical PhD fellowships 102466/Z/13/A [to A. B.] and 102464/Z/13/A [to L. P.], and the MLW Programme Core Award).

***Potential conflicts of interest.*** N. A. C. has received research grant support and honoraria from GlaxoSmithKline Biologicals for participation in rotavirus vaccine data safety monitoring board meetings. M. I.-G., K. C. J., N. B.-Z., and D. H. have received research grant support from GlaxoSmithKline Biologicals. M. I.-G. and D. H. have received research grant support from Sanofi Pasteur MSD/Merck Sharp and Dohme. All other authors report no potential conflicts. All authors have submitted the ICMJE Form for Disclosure of Potential Conflicts of Interest. Conflicts that the editors consider relevant to the content of the manuscript have been disclosed.
