# Supplementary material for: Etiology of Diarrhea Among Hospitalized Children in Blantyre, Malawi, Following Rotavirus Vaccine Introduction: A Case-Control Study
Source: J Infect Dis. 2019 Feb 28;220(2):213–8. doi: 10.1093/infdis/jiz084 (PMC6581894; doi:10.1093/infdis/jiz084)
Supplement: jiz084_suppl_Supplementary_Figure_1 [file jiz084_suppl_supplementary_figure_1.docx]

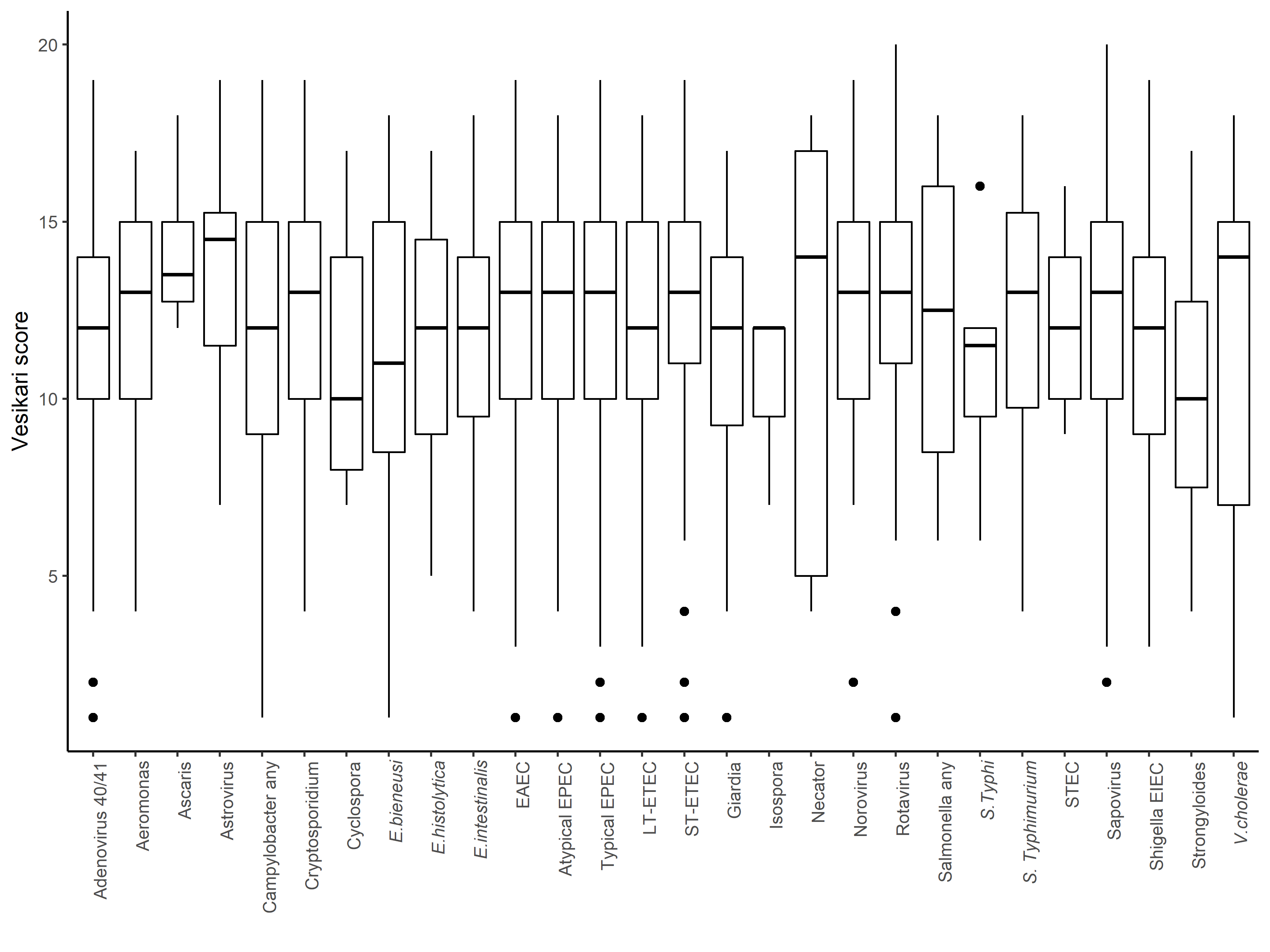


**Supplementary Figure 1: Vesikari scores among hospitalised diarrhoea cases by pathogen detected, including mixed infections.** In this plot, the bold horizontal line within the boxes represents the median value, while the lower and upper box edge correspond to the first and third quartiles (the 25th and 75th percentiles). The upper whisker extends from the box edge to the largest value no further than 1.5 * inter-quartile range (IQR) from the box edge (where IQR is the inter-quartile range, or distance between the first and third quartiles). The lower whisker extends from the box edge to the smallest value at most 1.5 * IQR of the box edge. Data points beyond the whiskers are called "outlying" points. EAEC=Enteroaggregative *E. coli*. EIEC=enteroinvasive *E.coli*. EPEC=enteropathogenic *E.coli*.LT-ETEC=heat-labile enterotoxin-producing *E.coli*. ST-ETEC=STh or STp-producing enterotoxigenic *E.coli*. STEC= shiga toxin-producing *E.coli*.
