# Supplementary material for: Etiology of Diarrhea Among Hospitalized Children in Blantyre, Malawi, Following Rotavirus Vaccine Introduction: A Case-Control Study
Source: J Infect Dis. 2019 Feb 28;220(2):213–8. doi: 10.1093/infdis/jiz084 (PMC6581894; doi:10.1093/infdis/jiz084)
Supplement: jiz084_suppl_Supplementary_Figure_2 [file jiz084_suppl_supplementary_figure_2.docx]

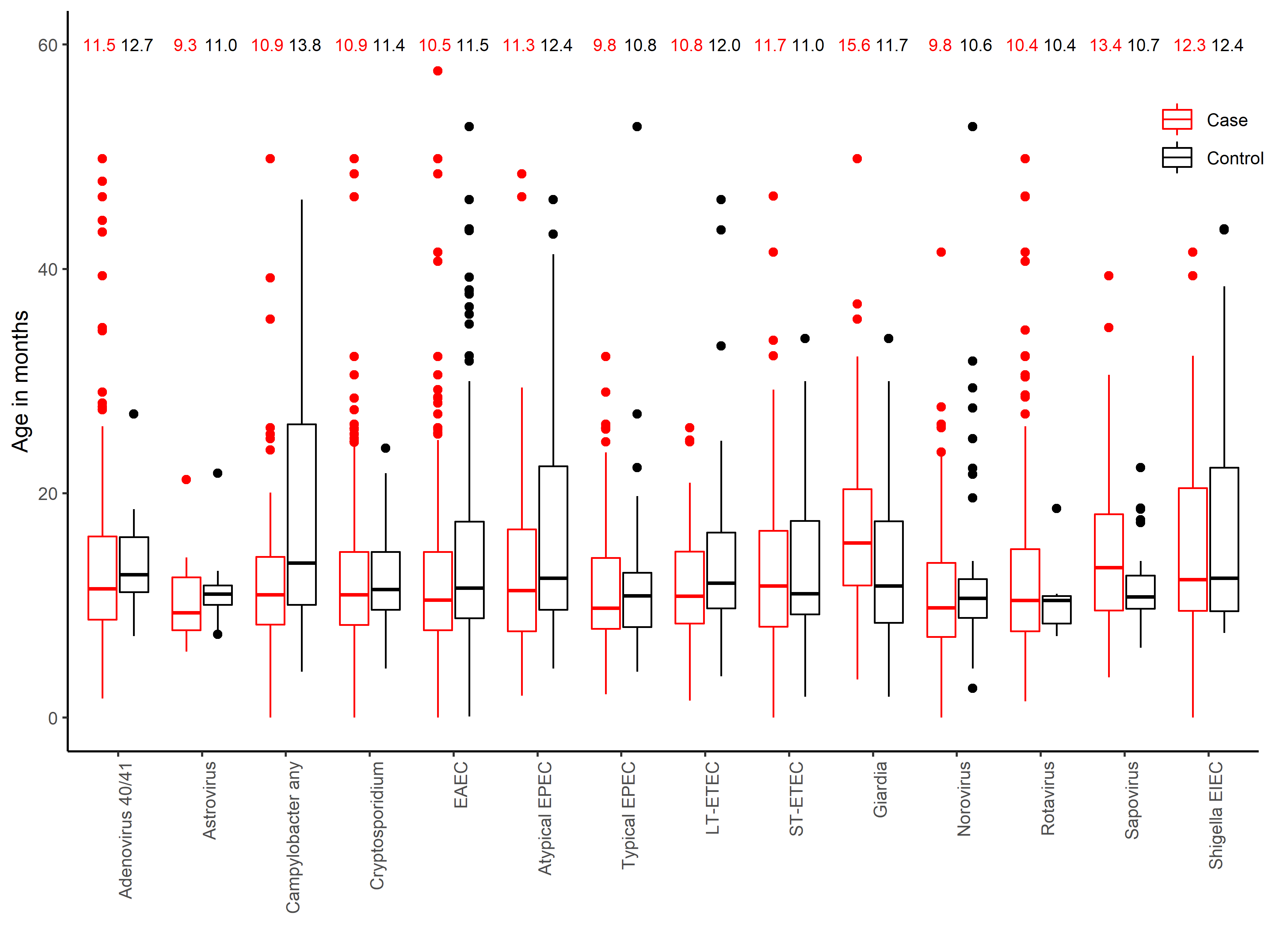


**Supplementary Figure 2: Age distribution by pathogen detected for hospitalised diarrhoea cases and asymptomatic community controls, including mixed infections.** Red boxplots correspond to cases, while the black boxplots correspond to controls. The bold horizontal line within the boxes represents the median value, the lower and upper box edge correspond to the first and third quartiles (the 25th and 75th percentiles). The upper whisker extends from the box edge to the largest value no further than 1.5 * inter-quartile range (IQR) from the box edge (where IQR is the inter-quartile range, or distance between the first and third quartiles). The lower whisker extends from the box edge to the smallest value at most 1.5 * IQR of the box edge. Data points beyond the whiskers are called "outlying" points. The median age of cases and controls (in months) is listed at the top. Plot includes only pathogens detected with a prevalence >5% in either cases or controls. EAEC=Enteroaggregative *E. coli*. EIEC=enteroinvasive *E.coli*. EPEC=enteropathogenic *E.coli*.LT-ETEC=heat-labile enterotoxin-producing *E.coli*. ST-ETEC=STh or STp-producing enterotoxigenic *E.coli*.
