# Supplementary material for: Etiology of Diarrhea Among Hospitalized Children in Blantyre, Malawi, Following Rotavirus Vaccine Introduction: A Case-Control Study
Source: J Infect Dis. 2019 Feb 28;220(2):213–8. doi: 10.1093/infdis/jiz084 (PMC6581894; doi:10.1093/infdis/jiz084)
Supplement: jiz084_suppl_Supplementary_Table_1 [file jiz084_suppl_supplementary_table_1.docx]

**Supplementary Table 1: Characteristics of hospitalised diarrhoea cases and asymptomatic community controls**

|  | Cases (n=684) | | Controls (n=527) | |
| --- | --- | --- | --- | --- |
| Characteristic |  | **Denominator** |  | **Denominator** |
| Age in months (median, IQR) | 10.7 (7.9-15.4) | 683 | 12 (9.3-18.7) | 521 |
| Female (n, %) | 299 (44%) | 684 | 270 (51%) | 526 |
| Middle-upper arm circumference (cm) |  | 677 |  | 527 |
| Median (IQR) | 13.0 (12.5-14.0) |  | 15.3 (14.0-16.5) |  |
| Severe acute malnutrition <11.5cm (n, %) | 60 (9%) |  | 2 (0.4%) |  |
| HIV^a^: exposed (n, %) | 121 (18%) | 665 | - | - |
| RV eligible (n, %) | 454 (66%) | 683 | 416 (80%) | 521 |
| RV coverage (n, %) | 406 (97%) | 419 | 366 (95%) | 385 |
| Vesikari score (median, IQR) | 13 (10-15) | 684 | NA |  |
| Recruitment year (n, %) |  | 684 |  | 527 |
| 2012 | 55 (8%) |  | 1 (0%) |  |
| 2013 | 228 (33%) |  | 108 (20%) |  |
| 2014 | 151 (22%) |  | 237 (45%) |  |
| 2015 | 250 (37%) |  | 134 (25%) |  |
| 2016 | 0 (0%) |  | 47 (9%) |  |
| Number of pathogens (n, %) |  | 684 |  | 527 |
| 0 | 43 (6%) |  | 137 (26%) |  |
| 1 | 78 (11%) |  | 42 (8%) |  |
| 2 | 82 (12%) |  | 95 (18%) |  |
| 3 | 127 (19%) |  | 105 (20%) |  |
| 4 or more | 354 (52%) |  | 148 (28%) |  |
| Mean (SD) | 3.65 (2.11) |  | 2.42 (1.96) |  |

^a^ HIV exposed is defined as a positive maternal HIV rapid test. For controls, maternal HIV status was only recorded for controls recruited through the RotaRITE study (n=105). SD= Standard Deviation.
