# Supplementary material for: Etiology of Diarrhea Among Hospitalized Children in Blantyre, Malawi, Following Rotavirus Vaccine Introduction: A Case-Control Study
Source: J Infect Dis. 2019 Feb 28;220(2):213–8. doi: 10.1093/infdis/jiz084 (PMC6581894; doi:10.1093/infdis/jiz084)
Supplement: jiz084_suppl_Supplementary_Table_2 [file jiz084_suppl_supplementary_table_2.docx]

**Supplementary Table 2: Prevalence of enteric pathogens in hospitalised diarrhoea cases and asymptomatic community controls by age group.** Pathogens are listed in order of decreasing prevalence among cases <12 months.

|  | **Age: <12 months** | | | **Age: 12-23 months** | | | **Age: 24-59 months** | | |
| --- | --- | --- | --- | --- | --- | --- | --- | --- | --- |
|  | **Cases** | **Controls** | **p-value** | **Cases** | **Controls** | **p-value** | **Cases** | **Controls** | **p-value** |
|  | **n=403 (%)** | **n=259 (%)** |  | **n=219 (%)** | **n=192 (%)** |  | **n=61 (%)** | **n=70 (%)** |  |
| **EAEC** | 216 (53.6) | 135 (52.1) | 0.771 | 116 (53) | 81 (42.2) | 0.037 | 22 (36.1) | 32 (45.7) | 0.347 |
| **Rotavirus** | 143 (35.5) | 6 (2.3) | <0.001 | 75 (34.2) | 1 (0.5) | <0.001 | 19 (31.1) | 0 (0) | <0.001 |
| **Adenovirus 40/41** | 108 (26.8) | 5 (1.9) | <0.001 | 75 (34.2) | 7 (3.6) | <0.001 | 16 (26.2) | 1 (1.4) | <0.001 |
| ***Cryptosporidium*** | 108 (26.8) | 24 (9.3) | <0.001 | 65 (29.7) | 17 (8.9) | <0.001 | 17 (27.9) | 1 (1.4) | <0.001 |
| **Typical EPEC** | 80 (19.9) | 27 (10.4) | 0.002 | 36 (16.4) | 14 (7.3) | 0.0074 | 7 (11.5) | 2 (2.9) | 0.081 |
| **ST-ETEC** | 75 (18.6) | 24 (9.3) | 0.002 | 57 (26) | 16 (8.3) | <0.001 | 13 (21.3) | 5 (7.1) | 0.036 |
| ***Campylobacter* any** | 62 (15.4) | 45 (17.4) | 0.568 | 44 (20.1) | 25 (13) | 0.0749 | 7 (11.5) | 31 (44.3) | <0.001 |
| **Norovirus** | 55 (13.6) | 32 (12.4) | 0.717 | 24 (11) | 8 (4.2) | 0.017 | 4 (6.6) | 5 (7.1) | 1 |
| **Shigella/EIEC** | 53 (13.2) | 13 (5) | 0.001 | 37 (16.9) | 9 (4.7) | <0.001 | 18 (29.5) | 7 (10) | 0.009 |
| **Atypical EPEC** | 41 (10.2) | 37 (14.3) | 0.139 | 27 (12.3) | 26 (13.5) | 0.827 | 8 (13.1) | 16 (22.9) | 0.226 |
| **LT-ETEC** | 40 (9.9) | 35 (13.5) | 0.195 | 25 (11.4) | 30 (15.6) | 0.269 | 3 (4.9) | 5 (7.1) | 0.723 |
| **Sapovirus** | 30 (7.4) | 23 (8.9) | 0.605 | 25 (11.4) | 11 (5.7) | 0.063 | 9 (14.8) | 0 (0) | <0.001 |
| ***Enterocytozoon bieneusi*** | 16 (4) | 15 (5.8) | 0.371 | 12 (5.5) | 4 (2.1) | 0.123 | 3 (4.9) | 1 (1.4) | 0.338 |
| ***Giardia*** | 13 (3.2) | 39 (15.1) | <0.001 | 27 (12.3) | 29 (15.1) | 0.500 | 10 (16.4) | 4 (5.7) | 0.086 |
| **Aeromonas** | 11 (2.7) | 4 (1.5) | 0.426 | 11 (5) | 6 (3.1) | 0.474 | 5 (8.2) | 0 (0) | 0.02 |
| **Salmonella any** | 10 (2.5) | 1 (0.4) | 0.058 | 12 (5.5) | 4 (2.1) | 0.123 | 8 (13.1) | 0 (0) | 0.0017 |
| **Cyclospora** | 9 (2.2) | 3 (1.2) | 0.383 | 4 (1.8) | 1 (0.5) | 0.377 | 0 (0) | 0 (0) | - |
| ***Encephalitozoon intestinalis*** | 8 (2) | 8 (3.1) | 0.520 | 6 (2.7) | 11 (5.7) | 0.204 | 1 (1.6) | 0 (0) | 0.466 |
| ***Entamoeba histolytica*** | 8 (2) | 5 (1.9) | 1 | 1 (0.5) | 3 (1.6) | 0.344 | 1 (1.6) | 0 (0) | 0.466 |
| ***Salmonella Typhimurium*** | 8 (2) | 1 (0.4) | 0.098 | 4 (1.8) | 0 (0) | 0.126 | 4 (6.6) | 1 (1.4) | 0.183 |
| **Astrovirus** | 7 (1.7) | 10 (3.9) | 0.152 | 5 (2.3) | 3 (1.6) | 0.729 | 0 (0) | 0 (0) | - |
| **Strongyloides** | 6 (1.5) | 2 (0.8) | 0.492 | 3 (1.4) | 4 (2.1) | 0.71 | 1 (1.6) | 1 (1.4) | 1 |
| **STEC** | 5 (1.2) | 0 (0) | 0.163 | 0 (0) | 1 (0.5) | 0.467 | 0 (0) | 0 (0) | - |
| **Ascaris** | 3 (0.7) | 1 (0.4) | 1 | 0 (0) | 3 (1.6) | 0.101 | 1 (1.6) | 1 (1.4) | 1 |
| **Necator** | 3 (0.7) | 1 (0.4) | 1 | 1 (0.5) | 0 (0) | 1 | 1 (1.6) | 0 (0) | 0.466 |
| **Isospora** | 2 (0.5) | 1 (0.4) | 1 | 1 (0.5) | 0 (0) | 1 | 0 (0) | 0 (0) | - |
| ***Salmonella Typhi*** | 2 (0.5) | 0 (0) | 0.523 | 2 (0.9) | 2 (1) | 1 | 4 (6.6) | 0 (0) | 0.046 |
| ***Vibrio cholerae*** | 2 (0.5) | 0 (0) | 0.523 | 3 (1.4) | 0 (0) | 0.251 | 4 (6.6) | 0 (0) | 0.045 |
| **Ancyclostoma** | 0 (0) | 0 (0) | - | 0 (0) | 0 (0) | - | 0 (0) | 0 (0) | - |
| ***Salmonella Enteritidis*** | 0 (0) | 0 (0) | - | 0 (0) | 0 (0) | - | 0 (0) | 0 (0) | - |
| **Trichuris** | 0 (0) | 0 (0) | - | 0 (0) | 1 (0.5) | 0.467 | 0 (0) | 0 (0) | - |

EAEC=Enteroaggregative *E. coli*; EIEC=enteroinvasive *E.coli*; EPEC=enteropathogenic *E.coli*.LT-ETEC=heat-labile enterotoxin-producing *E.coli*; ST-ETEC=STh or STp-producing enterotoxigenic *E.coli*; STEC= shiga toxin-producing *E.coli*.
